# An Overview of Metabolomic Approaches to Polyphenol Profiling for Nutraceutical Development

**DOI:** 10.3390/molecules31091468

**Published:** 2026-04-28

**Authors:** Temitope Oluwaferanmi Egbeniyi, Julius Dongsogo, Titilayo Oluwayemisi Bamidele, Alberta N. A. Aryee

**Affiliations:** 1 Department of Food Technology, University of Ibadan, P.M.B. 22133, Ibadan 200284, Oyo State, Nigeria; 2 Department of Biochemistry, Faculty of Biosciences, University of Development Studies, Tamale NT-0272-1946, Ghana; jdongsogo@gmail.com; 3 Department of Biochemistry, Nasarawa State University, P.M.B. 1022, Keffi 961101, Nasarawa State, Nigeria; 4 Food Science & Biotechnology Program, Department of Human Ecology, College of Agriculture, Science and Technology, Delaware State University, 1200 N DuPont Highway, Dover, DE 19901, USA

**Keywords:** phytochemicals, environmental stressors, nutraceuticals, metabolomics, data analysis

## Abstract

Secondary plant metabolites such as polyphenols (flavonoids, phenolic acids, stilbenes, and lignans) are valued for their numerous benefits and commonly associated with antioxidants, anti-inflammatory, anticancer, neuroprotective, and antidiabetic effects. Comprehensive profiling facilitates their identification and quantification, with metabolomics emerging as an increasingly valuable tool. This current work provides an overview of recent application of metabolomics for investigating polyphenols with nutraceutical potential. It also highlights the influence of plant species and environmental stressors (both biotics and abiotic) inducing metabolic shifts that promote the production and accumulation of these bioactive compounds (BACs). While various analytical tools including mass spectrometry (MS) coupled with liquid chromatography (LC-MS) or gas chromatography (GC-MS), as well as nuclear magnetic resonance (NMR) spectroscopy have been utilized to identify the diverse group of polyphenol metabolites, LC-MS has been predominantly used due to its superior sensitivity and wider metabolite coverage, with flavonoids being the main compounds identified. The integration of bioinformatic tools and pathway enrichment analysis in metabolomics is providing expansive insight into the pool of polyphenols, and their bio-functional interpretation and metabolic variations beyond the narrow scope of chromatographic separation alone. This overview also identifies limitations of current methods and suggests directions for future research, aimed at facilitating the development of nutraceuticals.

## 1. Introduction

**Figure 1:**
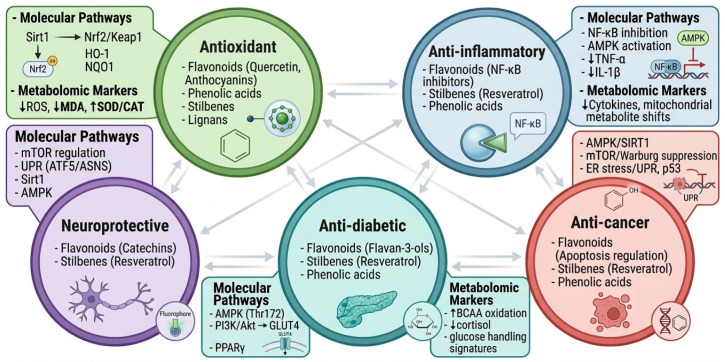
Functional spectrum of polyphenols highlighting interplay between antioxidant, anti-inflammatory, neuroprotective, antidiabetic and anticancer activities with specific classes for each polyphenol and their metabolic pathway involved. Created in SciSpace Biomedical, https://scispace.com/biomedical, accessed on 3 April 2026.

Polyphenols are a structurally diverse group of secondary plant metabolites characterized by one or more hydroxylated aromatic rings. They include flavonoids (flavonols, isoflavones, flavones, anthocyanins, proanthocyanins etc.), phenolic acids, lignans, stilbenes, and polyphenolic amides [1]. In plants these compounds play important roles in growth, metabolism, defense, and stress adaptation, and their synthesis is often upregulated under environmental stress conditions such as salinity [2,3,4,5]. Beyond their physiological roles in plants, polyphenols are widely recognized for antioxidant, antidiabetic, anti-inflammatory, and other health-promoting properties [6,7,8] (Figure 1).

**Figure 2:**
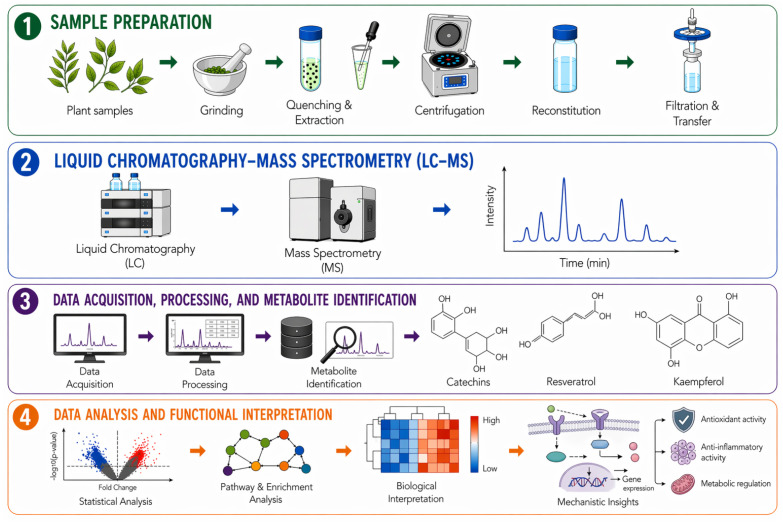
Workflow of liquid chromatography-mass spectrometry in metabolomics [16,22]. Created in SciSpace Biomedical, https://scispace.com/biomedical, accessed on 22 April 2026.

For instance, *Rhizobium rhizogenes*-transformed roots have been identified as rich sources of flavonoids, anthocyanins, proanthocyanidins, stilbenoids, and hydrolyzable tannins [9]. In addition, several indigenous and traditional plant foods including specialty rice (India), *Malus trilobata* (Lebanon), *Ximenia caffra* Sond. (South Africa), *Kigela africana* (Ghana), and *Canarium schweinfurthii* (Nigeria, Tanzania, Angola etc.), are recognized sources of bioactive compounds (BACs), necessitating their accurate and comprehensive characterization to establish their nutraceutical value [10,11,12,13,14]. Phytochemical profiling also aids in elucidating the regulatory mechanisms governing polyphenol accumulation in plants [15]. Among plants from different countries and regions, tea (*Camellia sinensis*), grapes (*Vitis vinifera*), and persimmons (*Diospyros kaki*) have demonstrated substantially high contents of polyphenols. Phytochemical identification relies primarily on chromatographic techniques such as thin-layer chromatography (TLC), column chromatography (CC), and liquid chromatography (LC), whereas structural elucidation is typically achieved using spectroscopic methods including MS, nuclear magnetic resonance (NMR) and infrared (IR) [16]. Because no single analytical platform provides complete metabolite coverage, hyphenated techniques such as LC-MS and LC-NMR are widely used to combine chromatographic separation with structural characterization (Figure 2).

Recent metabolomic analyses using liquid chromatography-orbitrap Fourier Transform mass spectrometry (LC-Orbitrap-FTMS) and gas chromatography time-of-flight mass spectrometry (GC-TOF-MS) identified more than 1400 metabolites in tea and showed that shading and developmental stages significantly alter the balance between galloylated catechins and amino acids [17]. In grapes, MS platforms such as direct infusion Fourier Transform ion cyclotron resonance mass spectrometry (DI-FT-ICR-MS) and reversed-phase ultra-high-performance liquid chromatography quadrupole time-of-flight mass spectrometry (RP-UHPLC-Q-ToF-MS) detected over 4500 compounds, particularly, anthocyanins and identified markers associated with smoke taint and water stress [18,19,20]. In persimmons, gallic acid and proanthocyanidin oligomers were identified as dominant polyphenols, with marked differences between astringent and non-astringent cultivars [11,21].

Metabolomics can be performed using untargeted approaches for broad metabolite profiling, or targeted approaches for quantification of predefined compounds [23]. Integration of both strategies enhances metabolite characterization, as demonstrated by Yang et al. [24]. Rapid advances in metabolomics has generated valuable insights into biotic and abiotic stress responses to polyphenol accumulation, processing-induced compositional changes, by-product valorization, and nutraceutical or therapeutic quality [3,4,15,24,25,26,27,28,29]. Given the structural complexity of polyphenols, advanced metabolomic workflows increasingly integrate bioinformatics, computational tools, statistical approaches to distinguish biologically meaningful associations from random variation [16,18,19,23,30,31,32] (Figure 2). These workflows support metabolite annotation and mapping to curated databases and ontologies, such as the Human Metabolome Database (HMDB), Gene Ontology (GO), and Kyoto Encyclopedia of Genes and Genomes (KEGG), as well as pathway enrichment tools such as MetaboAnalyst 6.0 including Mummichog, and metabolomics pathway analysis (MetPA). Collectively, these tools improve data processing, analytical sensitivity, annotation accuracy, and biological interpretation, thereby advancing comprehensive polyphenol profiling in complex biological matrices. However, substantial knowledge gaps remain. These include incomplete understanding of polyphenols biosynthesis and accumulation, limited resolution of stress-induced upregulation and downregulation patterns, insufficient holistic characterization of polyphenol profiles across complex plant matrices, and weak mechanistic linkage between polyphenol composition and bioactivity. In addition, bioinformatic interpretation remains underutilized relative to compound identification and quantification, while methodological variability, compound instability, incomplete annotation, and high analytical cost continue to limit precise and scalable profiling [22,33].

The significance of this review lies in its potential to provide a framework for integrating metabolomics within broader multi-omics approaches to polyphenol research across diverse plant matrices. Such an approach can strengthen mapping of metabolites to biological pathways and bioactivities. Improved understanding of stress-responsive biosynthesis and varietal differences may advance knowledge of polyphenol biosynthesis, compositional complexity, and bioavailability, thereby expanding opportunities for nutraceutical development and human health applications. This review therefore synthesizes current knowledge across plant matrices, compares analytical platforms, examines and discusses metabolomics data-analysis tools spanning data processing, statistical analysis, and functional interpretation. It also highlights methodological gaps and outlines future directions in advancing polyphenol research and nutraceutical development.

### Literature Selection and Scope

This review applied a structured literature search to identify studies on the metabolomics of polyphenols with nutraceutical potential. Peer-reviewed articles published between 2020 and 2025 were retrieved from PubMed and Google Scholar using keywords and Boolean operators, including “metabolomic platforms”, “plants”, “polyphenols”, “nutraceutical properties”, “environmental stressors AND polyphenol accumulation”, and “metabolomics data analysis”. Retrieved records were de-duplicated and screened in two stages: titles/abstracts followed by full-text assessment. Eligibility was defined by predefined criteria: included studies were (i) English-language original research (2020–2025), (ii) focused on metabolomics of plant-derived polyphenols, (iii) examined environmental stress effects on polyphenols accumulation, including biochemical changes (upregulation or downregulation), (iv) established links between polyphenols and bioactivities (antioxidant, anti-inflammatory, anticancer, neuroprotective, antidiabetic), and (v) addressed metabolomic data analysis and interpretation. Excluded were review articles, meta-analyses, books, conference abstracts, non-English publications, studies limited to genomics or transcriptomics without metabolomics data, research on non-plant-derived polyphenols, and articles without accessible full texts.

## 2. Bioactivities of Polyphenols

The bioactives of polyphenols include antioxidant, anti-inflammatory, neuroprotective, antidiabetic and anticancer benefits [15,34,35,36,37,38,39,40,41,42], Figure 1 and Table 1. They function by neutralizing ROS, reducing DNA mutations, and modulating enzymes and signaling pathways to reduce oxidative stress, inflammation and risk of chronic diseases. Brown seaweeds (e.g., *Sargassum asperum*) with high phenolic and flavonoid contents demonstrated strong antioxidant and reducing capacities, with metabolomics revealing compound-specific contributions [43]. Similarly, *Felicia abyssinica* extracts rich in quercetin-rutinoside and sinapate derivatives exhibited potent radical-scavenging activity, supported by metabolomic identification of oxygenated phenolics as key contributors [44].

**Figure 3:**
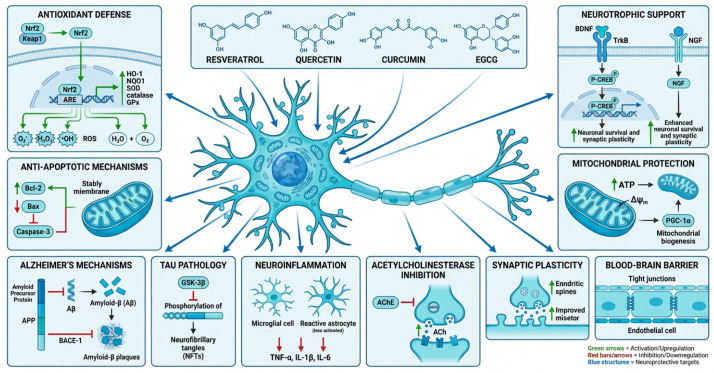
Potential mechanism of anti-inflammatory properties polyphenols. NF-κB↓ blocks IκB degradation → ↓p65 nuclear translocation, ↓IL-6, TNF-α, COX-2 suppresses major pro-inflammatory mediators, ↓p38/JNK/ERK → ↓AP-1 blocks activator protein-1 transcription factor activation, Scavenge ROS → ↓NLRP3 prevents inflammasome activation, ↓COX-2/LOX/iNOS → ↓PGE2/LTB4/NO reduces eicosanoid/nitric oxide production, ↓IL-1β/IL-6/TNF-α secretion, ↑IL-10 shifts cytokine balance toward resolution, ↓M1 → M2 promotes anti-inflammatory macrophage polarization and ↓T-cell activation reduces adaptive immune overactivation [15,45,52]. Created in SciSpace Biomedical https://scispace.com/biomedical, accessed on 3 April 2026.

Flavonoids and related metabolites identified using LC-MS have been shown to downregulate inflammatory mediators such as TNF-alpha and IL-6 in both cellular and animal models (Figure 3). Mulberry (*Morus alba* L.) leaf flavonoids, comprising ~30% flavonoid glycosides including quercetin and kaempferol derivatives, reduced cytokine secretion and suppressed inflammatory pathways in DSS-induced colitis mice [45]. UPLC-MS profiling of *Ludwigia adscendens*, an aquatic herb belonging to family Onagraceae and widely distributed in canals and drains in the Nile Delta, Egypt, identified gallic acid, quercetin, ellagic acid, and betulinic acid with antioxidant and anti-inflammatory properties [54]. Similarly, UPLC-MS/MS analysis of *Bienertia cycloptera* fractions identified 62 metabolites, including flavonoids and phenolic acids associated with anti- inflammatory activity [52]. The anti-inflammatory properties of the key phytochemicals (kaempferol-3-O-robinoside-7-O-rhamnoside, soyasaponins I and III, and 16- hydroxyhexadecanoic acid) identified in the leaf extract of *Gliricidia sepium* (Jacq.) Kunth ex Walp. through UHPLC-QTOF-MS/MS analysis have been linked to diabetic nephropathy [56].

Polyphenols exert anticancer effects through multiple mechanisms including, apoptosis induction (Bax↑/Bcl-2↓, caspase activation), cell cycle arrest (p53/p21↑, cyclin/CDK↓), ROS scavenging (Nrf2↑), anti-proliferation (PI3K/Akt/mTOR↓), suppression of angiogenesis (VEGF↓), metastasis inhibition (EMT↓), anti-inflammation (NF-κB↓), epigenetic modulation (HDAC↓), and chemosensitization [57,58,59]. UPLC-IMS-QTOF-MS analysis of *Garcinia subfalcata* identified 124 compounds, predominantly xanthones, flavonoids phloroglucinols [60]. UHPLC-QTOF-MS/MS characterized flavonoid glycosides and related phytoconstituents in *Dicliptera bupleuroides* with relevance to breast cancer therapy [46]. In *Annona muricata*, UHPLC-Orbitrap- HRMS identified 35 metabolites, including alkaloids, flavonoids, and acetogenins linked to anticancer potential [48]. Untargeted UHPLC-Q-TOF-MS^2^ profiling of fermented *Perilla frutescens*, an edible and medicinal plant grown in many East Asian countries quantified phytoconstituents with anticancer potential [47]. LC-MS profiling of *Selaginella* species revealed biflavonoids such as robustaflavone derivatives with significant cytotoxicity against cancer cell lines [61].

Phenolic acids and flavonoids demonstrate neuroprotective effects by mitigating oxidative stress and neuroinflammation implicated in disorders such as Parkinson’s and Alzheimer’s diseases. Mechanisms include suppression of ROS, inflammatory mediators, apoptosis, and modulation of the phosphoinositide 3-kinases/serine/threonine protein kinase (PI3K/AKT) pathway, and amelioration of STZ-induced neuroinflammation and amyloidogenesis [40,42,49]. GC-MS and LC-QTOF-MS/MS analyses of quinoa grains identified flavonoid glycosides correlated with anti-Alzheimer activity [62]. LC-MS profiling of *Hibiscus sabdariffa* detected anthocyanins and phenolic acids with anti-amyloidogenic, anti-inflammatory, antioxidant, and anti-acetylcholinesterase activities [40]. UPLC-MS/MS and chemometric approaches identified neuroprotective metabolites in Wen-Shen-Yang-Gan decoction [42]. LC-HR-ESI-MS analysis of *Ulva* sp. confirmed mitigation of neurodegeneration via PI3K/Akt pathway modulation through the suppression of elevated levels of tumor necrosis factor-α (TNF-α), interleukin-1β (IL-1β), and IL-6 together with the inhibition of ROS generation, apoptosis, inflammatory mediators, and the phosphoinositide 3-kinases/serine/threonine protein kinase (PI3K/AKT) pathway [49]. Additionally, UPLC/MS/MS and HPLC identified flavonoids (7-trihydroxyflavone, isorhainetin, vitexin, and apigenin) in *Citrus aurantium* extracts that protected neurons by regulating ROS and Akt-mediated CREB/BDNF (for neuroprotection) and GSK3β/NF-κB (for anti-inflammatory effects) pathways [50].

Phenolic acids such as chlorogenic and caffeic acids reduce glucose absorption and enhance insulin signaling in diabetic models, effects amplified in extracts from salinity-stressed plants [7]. Additional mechanisms include inhibition of advanced glycation end products (AGEs) formation and activation of PPARγ, improving lipid and glucose metabolism improving insulin sensitivity [51,55]. UHPLC-TOF-MS/MS profiling of red *Opuntia ficus-indica* fruit extracts revealed polyphenols with antidiabetic and anti-hypercholesterolemic properties [51]. Similarly, UHPLC-QTOF-MS/MS characterization of red cabbage and broccoli sprouts identified 24 metabolites, predominantly phenolics and amino acids, associated with enhanced antidiabetic potential following germination [55].

Beyond bioactivity screening, metabolomics enables mapping of biotransformation and gut microbiome interactions [40,42,45,52,57,58,59,63,64]. For instance, gallic acid supplementation reduced serum triglycerides, fat digestibility, and the Bacteroidetes/Firmicutes ratio in dogs, indicating modulation of lipid metabolism. Such approaches link metabolic signatures to health outcomes and validate mechanisms observed in vitro via in vivo models. Additionally, metabolomics supports quality control and safety evaluation of plant-derived products. Long-term gallic acid exposure was assessed using UPLC-Orbitrap-MS and multivariate analyses such as variable importance projection (VIP) [64], while UHPLC-Orbitrap HRMS has been applied to monitor safety and compositional changes in aged foods [39]. Overall, metabolomics offers a thorough mapping of polyphenol composition to functional efficacy, strengthening their application in nutraceutical development.

## 3. Biosynthesis, Accumulation and Diversity of Polyphenols

Polyphenols are broadly classified into four major groups: phenolic acids, flavonoids, stilbenes, and lignans [1,35]. Their biosynthesis in plants mainly involves the shikimate, phenylpropanoid, and aceto-malonate pathways. In polyphenolic compounds containing A and B aromatic rings, whereas the A-ring is formed through the aceto-malonate pathway [8]. These pathways are metabolically linked: the shikimate pathway produces aromatic amino acids precursors, the phenylpropanoid pathway generates intermediates such as *p*-coumaroyl-CoA, and the aceto-malonate pathway uses these intermediates to form additional ring structures and derivatives. In the shikimate pathway, shikimic acid is converted into aromatic amino acids, principally L-phenylalanine and L-tyrosine, which are then deaminated by phenylalanine ammonia lyase (PAL) to form trans-cinnamic acid [43,45,46]. This intermediate enters the phenylpropanoid pathway, which leads to the synthesis of phenolic compounds and related derivatives, including flavonoids, lignins, coumarins, and stilbenes. In the aceto-malonate pathway, malonyl-CoA condenses with p-coumaroyl-CoA via chalcone synthase to produce chalcone, a key intermediate in flavonoid biosynthesis [1,7,9,43,45,46]. Additionally, through this pathway, carbon derived from carbohydrate, amino acid, and lipid metabolism is integrated into the biosynthesis of flavonoids. The polyphenolic metabolites produced through these coordinated pathways contribute to plant defense and stress adaptation and are widely associated with antioxidant, anti-inflammatory, and anticancer bioactivities.

Bioactive polyphenols such as quercetin, kaempferol, catechin, chlorogenic acid, and resveratrol have been identified in various plants [15,34,35,36,37,38,39,41]. Metabolomic analyses have further identified novel glycosylated and acylated derivatives that enhance or modulate these bioactivities. Among plant metabolomic analyses, flavonoids and simple phenolic acids are the predominant polyphenols particularly flavonoid glycosides (flavonols, flavones, anthocyanins, etc.) and hydroxycinnamic/hydroxybenzoic acids were the most frequently detected with minor yet significant representation of stilbenoids, lignans, coumarins and complex tannins [7]. Although traditional in vitro assays show positive correlations between total phenolic content and antioxidant capacity [65], integrating metabolomics extends these findings by elucidating metabolic fate and revealing mechanistic pathways.

### Impact of Environmental Stressors on Polyphenol Accumulation

**Figure 4:**
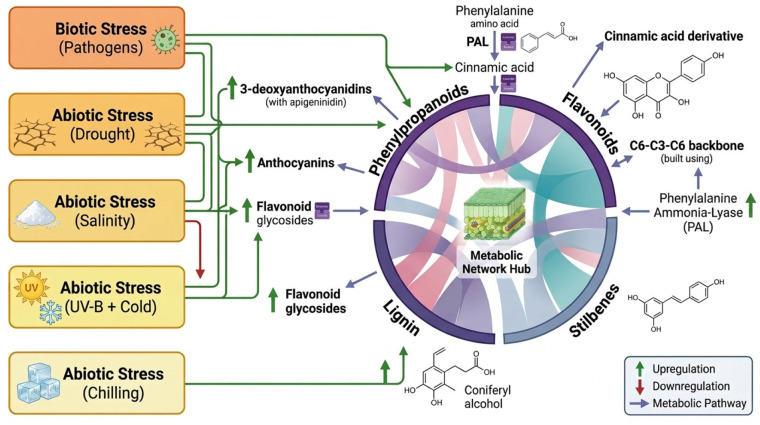
Integrated network of polyphenols, visualizing metabolic shifts; upregulation or downregulation of polyphenols due to environmental stress [2,4,24,27]. Created in SciSpace Biomedical, https://scispace.com/biomedical, accessed on 3 April 2026.

Functional foods derived from stress-enhanced plants represent a promising strategy for improving nutraceutical value. However, the optimization of polyphenol accumulation and associated bioactivity requires controlled environmental modulation, since abiotic stresses such as temperature extremes, drought, flooding, light intensity, salinity, and heavy metals can alter agricultural productivity, polyphenol biosynthesis, and overall nutraceutical quality [2,4,5,25,27,33,66] (Figure 2). In addition, interspecies and cultivar-dependent variation contributes to differences in polyphenol composition, while postharvest processing such as fermentation and drying further influences metabolite stability and bioavailability [24,63,67]. Numerous studies have shown that abiotic stress can stimulate the accumulation of polyphenols, particularly phenylpropanoid pathway-derived compounds such as phenolic acids, flavonoids, stilbenoids, and lignans [3,4,5,8,25,27,33,35,41,66,68,69] (Figure 4). These responses are generally mediated by reactive oxygen species (ROS), which activate key biosynthetic enzymes such as PAL, chalcone synthase, and stilbene synthase. However, the response is typically biphasic: low to moderate stress enhances phenolic accumulation as a defense mechanism, whereas severe or prolonged stress suppresses biosynthesis or promotes compound degradation [3,4,25,27,33,35,66]. Specific stress conditions have been associated with distinct polyphenolic responses. Low temperature exposure has been associated with increased accumulation of tannins and phenolic acids, while salinity has been reported to enhance the synthesis of phenolic acids, flavonoids, hydroxycinnamic acids, anthocyanins, and chlorogenic acid, although anthocyanin responses are often species-dependent [2,3,8,25,27,33,35,66]. Moderate drought has also been shown to increase caffeic and p-coumaric acid contents in grape must and wine [70], whereas shading in tea shoots reduces the proportion of galloylated catechins, with the effect being more pronounced in fully matured leaves than in young shoots [17]. Metabolomic analysis of the halophyte *Halogeton glomeratus* identified 2152 metabolites, including flavones, and flavonols consistent with its adaptation to salinity, drought, and heavy metal stress [68]. Similarly, *C. deserticola* callus cultures have been proposed as sustainable and controlled production systems for high-value secondary metabolites, underscoring the biotechnological potential of stress-adapted wild plants [5]. Stilbenes, particularly resveratrol, are commonly induced under stress conditions and are widely recognized for cardioprotective and anticancer activities [57,58]. Furthermore, lignans, produced through oxidative coupling of monolignols, contribute to plant structural defense and have also been associated with neuroprotective effects in humans [6,42].

## 4. Metabolomic Approaches

Metabolomics plays a central role in mapping polyphenol profiles to bioefficacy by providing quantitative and molecular-level insights into mechanisms of action and predicting therapeutic potential [38]. A wide range of plant materials, including medicinal herbs, fruits, and crops from diverse geographical regions, have been investigated using metabolomic techniques to characterize polyphenols and evaluate their nutraceutical potential (Table 2). Among these, LC-MS is the most frequently applied platform for broad characterization due to its sensitivity and ability to elucidate structures of diverse polyphenols [19,31]. GC-MS is particularly suited for volatile and thermally stable metabolites but is less appropriate for high-molecular-weight polyphenols or complemented with NMR for structural confirmation and quantitative analysis without derivatization. Coupling UHPLC with high-resolution MS (QTOF or Orbitrap) enhances mass accuracy and metabolite coverage, enabling detection of diverse polyphenols including glycosylated and methylated derivatives [36,41,46,55]. Spatial LC-MS profiling of Tartary buckwheat achenes identified 17 phenolic acids and 83 flavonoids, alongside stilbenoids, lignans, and tannins [9,71]. LC-MS analysis identified kaempferol quercetin, kaempferol, and their derivatives as the main flavonoids in mulberry leaf extracts [45]. HPLC-photodiode array detection and headspace-solid phase microextraction (HS-SPME)/GC-MS analyses of *Eleutherococcus senticosus* (Rupr. et Maxim.) fruits identified eleutherosides B, E, and E1 in addition to phenolic acids [72].

UPLC-MS combined with supervised machine learning (ML) to differentiated soybean varieties and cultivation sites and identified 31 phenolic compounds, predominantly isoflavones and quercetin derivatives [79]. LC-HR-ESI-MS profiling of *Bignonia binata* leaves identified phenylethanoids, flavonoid glycosides, and iridoids associated with hepatoprotective and nephroprotective effects in carbon tetrachloride (CCl4)-intoxicated rats [74]. Similarly, UHPLC-QTOF-MS/MS studies showed enhanced antidiabetic metabolites following germination [55], while analysis of *Cydonia oblonga* (quince) identified anthocyanins, flavan-3-ols, flavonols, hydroxycinnamic and hydroxybenzoic acids, lignans, stilbenes, and low-molecular-weight phenolics [38]. UHPLC-QTOF-MS/MS has also been used to characterized polyphenols in traditional formulations such as Shuang Huang Lian, identifying 17 flavonoids among other components [75]. Furthermore, UHPLC-MS/MS analysis of *Gliricidia sepium* (Jacq.) Kunth. ex Walp leaf extract identified four major constituents, including kaempferol-3-O-robinoside-7-O-rhamnoside, soyasaponin I & III, and 16-hydroxyhexadecanoic acid [56].

UHPLC-Q-Exactive Orbitrap MS and air-flow-assisted desorption electrospray ionization MS imaging analyses of *Cocculus orbiculatus*, a medicinal herb valued for anti-inflammatory, analgesic, diuretic, and other therapeutic properties revealed tissue-specific distribution of alkaloids and flavonoids, with higher content found in the roots than the stem and flower [76]. Additional applications include UHPLC-Orbitrap HRMS profiling of polyphenols and anthocyanins in aged black garlic [39], identifying cinnamic acids, phenolic acids derived from galloyl quinic and shikimic acid, proanthocyanidins, glycosylated flavonoids, triterpenes and other phenols and 81 compounds in *Serjania marginata* via UHPLC-ESI-HRMS and NMR spectroscopy [31], and detecting anticancer-related phenolic compounds such as apigenin, p-coumaric acid, rosmarinic acid, caffeic acid, polygallic acid, phenprobamate, hydroxy acetophenone, allopurinol, homovanillic acid, danshensu, and N-malayamycin in fermented *Perilla frutescens* using UHPLC-Q-TOF-MS^2^ [47].

Targeted HPLC-based methods remain valuable. HPLC coupled with ultraviolet (UV)/diode array detection (DAD) enabled simultaneous quantification of flavonoids (quercetin, kaempferol, catechin, hesperetin, naringenin, hesperidin, and naringin), cinnamic acid derivatives (p-coumaric acid, ferulic acid, and caffeic acid), and benzoic acids (vanillic acid and 4-hydroxybenzoic acid) in the leaves and inflorescences of *Amaranthus cruentus*, demonstrating antioxidant, antidiabetic, and antihypertensive activities [78]. Reversed-phase UHPLC-DAD identified 69 phenolic compounds in plant foods relevant to nutraceutical applications [80]. HPLC-DAD-ESI-IT-TOF-MS^n^ characterized bioactive polyphenols in *Astragali radix* [77], while HPLC-ESI-TOF-MS highlighted solvent- and tissue-dependent variations in antioxidant and antimicrobial activity of *Uapaca togoensis* leaves and stem bark [81]. UHPLC-HRMS profiling of *Phlomis* species established distinct chemical fingerprints relevant to nutraceutical formulation [65], and UHPLC-MS identified neuroprotective polyphenols with potential relevance to Parkinson’s disease [42]. Furthermore, walnut polyphenols analyzed via HPLC high-resolution Fourier transform MS (HPLC-HRFTMS) demonstrated inhibition of starch-digesting enzymes and intestinal glucose transport [7].

GC-MS remains suitable for volatile or derivatized metabolites. For example, GC-MS and FT-IR analysis of *Aporosa cardiosperma* detected substantial flavonoid, phenol, and tannin contents [29], while untargeted GC-MS analysis of *Combretum platypetalum* identified 17 BACs, including flavonoids and terpenoids [28]. Combined GC-MS and LC-MS profiling of *Cajanus scarabaeoides* revealed flavonoids and polyphenols as dominant bioactives [73]. Moreover, NMR offers non-destructive analysis, structural validation, and absolute quantification. When combined with UPLC-MS/MS, NMR enabled comprehensive profiling of *Crescentia cujete* fruit pulp, identifying n-alkyl glycosides, phenolic acid derivatives, flavonoids, phenylethanoids, and iridoid glycosides [19]. For instance, *Felicia abyssinica* extracts were analyzed using GC-MS for derivatized polyphenol fractions and fatty acid methyl esters, LC-ESI-MS/MS for flavonoids and phenolics, and 1D/2D NMR for structural confirmation [44]. Additionally, matrix-assisted laser desorption/ionization MS imaging (MALDI-MSI) has emerged as a powerful tool for mapping spatial metabolite distribution within plant tissues [71].

The different metabolomic approaches presents distinct advantages and drawbacks that affect their suitability and application (Table 3). Various analytical platforms resolves the complexities of diverse plant matrices with non-volatile, polar, thermally stable and unstable phenolic compounds presenting various levels of precision, sensitivity, and selectivity for structural identification and fingerprinting. For instance, although high-resolution MS can detect numerous features, only a small fraction are confidently identified, highlights the need for advancements in the development of integrated MS/MS and NMR spectral libraries [82] and other tools [83].

### 4.1. Data Analysis and Interpretation

**Figure 5:**
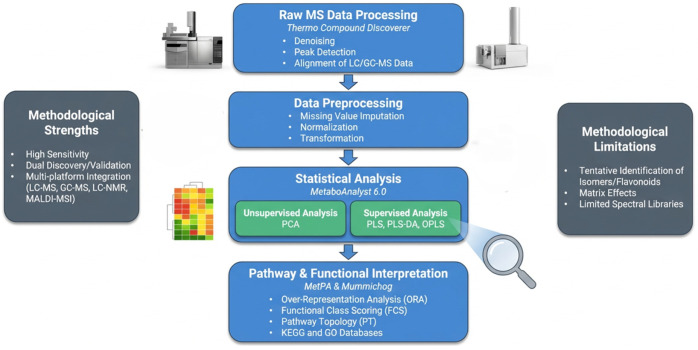
Data analysis and interpretation of polyphenol metabolomics. Created in SciSpace Biomedical, https://scispace.com/biomedical, accessed on 3 April 2026.

Metabolomics datasets can be analyzed using platforms such as MetaboAnalyst 6.0 and Thermo Compound Discoverer, the latter being specialized for raw LC/GC-MS processing (Figure 5). MetaboAnalyst 6.0 is a web-based platform that supports data processing, statistical analysis, visualization, pathway enrichment analysis, biomarker discovery, network analysis, power analysis, and integration with other omics datasets [81]. Although initially developed for targeted metabolomics, it now supports both quantitative and untargeted workflows and includes modules for MS/MS spectral processing, compound annotation, dose-response analysis, and integration of metabolite genome-wide association studies with Mendelian randomization for causal inference.

Interpretation of metabolomics data generally follows a structured workflow consisting of four major stages: raw spectral preprocessing, peak area preprocessing, statistical analysis, and pathway enrichment [84]. Spectral preprocessing includes denoising, peak detection and alignment, and metabolite identification, followed by missing value imputation, data transformation to correct skewed distributions, and normalization to reduce technical variability. Statistical analysis then applies univariate and/or multivariate methods to identify significantly discriminant metabolites and characterize relationships within the dataset. Multivariate methods are particularly valuable in metabolomics because they enable simultaneous analysis of multiple variables in high-dimensional datasets. Principal component analysis (PCA), an unsupervised method, is commonly used for dimensionality reduction and visualization of sample clustering patterns, as shown by Zhang et al. [23] in differentiating metabolic phenotypes among three *Camellia* species. In contrast, partial least squares (PLS), partial least squares discriminant analysis (PLS-DA) are supervised methods widely used for classification of samples into predefined groups, in high-dimensional, collinear datasets such as LC-MS and NMR data, while orthogonal projections to latent structures (OPLS), further improves interpretability by separating predictive variation from orthogonal, from non-predictive variation [84]. These analytical approaches enable robust interpretation and biological contextualization of metabolomics datasets. For instance, Farazi et al. [7] used Thermo Compound Discoverer (v.3.3) for raw data processing, while Manickam et al. [26] highlighted the value of multivariate analysis for identifying metabolite patterns associated with specific biological factors. A summary of statistical analyses and pathway-based tools used for the functional interpretation of metabolomics data is presented in Table 4.

#### 4.1.1. Pathway Enrichment Analysis

Pathway enrichment analysis is critical for interpreting large and complex metabolomics datasets, particularly those generated by LC-MS platforms [30,85]. It enables the integration of detected metabolites with biological functions through pathway mapping tools such as MetPA, Metscape, and Mummichog (Table 4). MetPA is commonly used to identify significantly perturbed pathways by combining over-representation analysis with pathway topology (PT) analysis. It typically requires processed peak lists or annotated compound identifiers, such as KEGG or HMDB, and generates pathway impact scores that help prioritize biologically relevant pathways [30,84]. In contrast, Mummichog is particularly useful in untargeted metabolomics because it maps mass-to-charge ratio (*m*/*z*) features directly onto metabolic networks without requiring prior metabolite identification [77]. By leveraging high-resolution MS data, it enables early functional interpretation of metabolomic profiles before formal compound annotation, making it particularly valuable for label-free untargeted workflows [82,84,85]. Since biological pathways consist of interconnected metabolites that regulate cellular processes, pathway enrichment analysis provides a systems-level framework of metabolic perturbations beyond individual metabolite changes.

Over-representation analysis (ORA) is among the most widely used approaches and evaluates whether significantly altered metabolites are enriched in predefined pathways more than expected by chance. Other enrichment strategies, including functional class scoring (FCS), GO analysis, KEGG pathway analysis, and PT analysis, are also widely applied to elucidate metabolic mechanisms and improve biological interpretation [84]. Several studies demonstrate the value of these approaches. Zhang et al. [23] used GO and KEGG enrichment analyses through the Metascape platform to identify 78 molecular targets linked to four therapeutic activities. Zhang et al. [38] applied multivariate statistical analysis to interpret metabolic signatures associated with nutritional potential in an untargeted UHPLC-QTOF-MS study. Ramabulana et al. [86] used KEGG identifiers to annotate differentially abundant metabolites in four *Momordica* species and found enrichment in glycerophospholipid metabolism and flavonoid biosynthesis, including key aglycones such as quercetin and kaempferol. Overall, pathway enrichment analysis are critical for translating raw metabolomic data into biological insight and for supporting the discovery of bioactive compounds, including polyphenol-derived nutraceuticals relevant to pharmaceutical applications [32,83]. The standardization of these methodologies has been highlighted by Wieder et al. [30], who provided recommendations for best practices.

#### 4.1.2. Data Requirements, Results Interpretation, and Application Scope

Advances in analytical tools and platforms are central to ensuring the validity, reproducibility, and utility of metabolomic analyses. Meaningful insights depend on properly preprocessed input data, statistical analyses, while pathway mapping, ML, and advanced computational tools strengthen prediction and biological interpretation (Table 5). Visualization approaches (heatmaps, PCA and PLS-DA plots, and network diagrams) further support pattern recognition and relationship mapping. Collectively, these approaches underpin applications in drug development, personalized medicine, disease diagnosis, and biomarker discovery.

### 4.2. Methodological Strengths and Gaps

Consumer demand for natural products has increased interest in metabolomics-guided identification of bioactive metabolites provides an important foundation for nutraceutical development. Contemporary workflows frequently integrate untargeted LC-MS with targeted quantification the detection and validation of polyphenols. In addition, spatial imaging techniques such as MALDI-MSI enable tissue-level localization, complementing quantitative LC-MS datasets [18,19,31,42,52,61,71,73,74,80]. Furthermore, NMR is non-destructive and enables structural confirmation and simultaneous quantification of multiple compounds in a single extract. Multi-platform integration combines complementary techniques, including GC-MS, LC-MS, LC-NMR, along with various omics technologies such as genomics, epigenomics, transcriptomics, and proteomics further expand view of biological processes Despite these analytical strengths, several methodological limitations remain. Compound identification is often tentative without authentic standards, especially for isomeric flavonoids and phenolic acids, and absolute quantification is constrained by matrix effects and methodological variability. Inconsistent sample preparation, extraction procedures, ionization conditions, reporting practices, limited spectral libraries, and annotation discrepancies reduce reproducibility and cross-study comparability, while variation in stress intensity and duration further complicates interpretation [82,83]. Greater transparency through reporting raw spectra, annotated peak lists, and adherence to metabolite identification standards is essential to improve confidence in metabolomics data [76]. In addition, the current emphasis on major crops and widely studied medicinal plants continues to overlook underutilized species with potentially unique stress-adaptive metabolites of nutraceutical and pharmacological relevance. Moreover, limited clinical validation of stress-induced bioactives continues to restrict translation of in vitro findings into human health application.

## 5. Future Directions

Metabolomic profiling provides a powerful toolset for uncovering dynamic metabolic adaptations of plants in response to stress that yield improved amounts of polyphenols, enabling the discovery and characterization of bioactive phytoconstituents for improved natural therapeutics. The translation of these insights into practical applications further emphasizes the need for comprehensive integration of omics data with functional bioassays and clinical trials. Future research should adopt multidimensional approaches integrating metabolomics with transcriptomics, proteomics, and bioassays to elucidate genomic regulation of polyphenol biosynthesis and bioactivity [87]. Furthermore, leveraging AI through ML, deep learning, and computer-aided structural elucidation will accelerate the identification, structural elucidation, and therapeutic profiling of phytochemical [83]. Advances in ML and network pharmacology can facilitate structural validation and chemical synthesis of these organic compounds to address the gap between demand and extraction capacity as well as non-food sources including vegetable waste, microalgae and algae [88,89]. Future investigations should also standardize metabolomic protocols, integrate with high-throughput bioactivity screening and clinical validation to strengthen translational impact. Advances in spatial-temporal and plant cell culture metabolomics offer promising tools for precision profiling and controlled production of polyphenols during plant development and stress progression for food and therapeutic ingredient development should be explored [90]. Furthermore, neglected indigenous plant species diversity should be studied in areas like Sub-Saharan Africa to uncover novel compounds relevant to nutraceuticals from stress-responsive plants and foster open data repositories through standardized reporting for metabolomic datasets to promote transparency and cross-study comparison. Standardization, methodological rigor, and broader species exploration will be critical to accelerate this translation from plant metabolic response to nutraceuticals, accelerating their development to address key health challenges facing society today and the future.

## 6. Conclusions

The expanding significance of natural products in health promotion calls for comprehensive assessment of bioactive compounds such as polyphenols. Although chromatography hyphenated with MS or NMR contribute valuable analytical information, no single technique is sufficient on its own. The integration of advanced metabolomics platforms has markedly propelled polyphenol research, enabling a more comprehensive and precise elucidation of these compounds in complex matrices. Furthermore, metabolomics effectively overcomes the constraints associated with conventional techniques by capturing the dynamic variability of polyphenols influenced by species, environmental stressors, and processing conditions. In parallel, bioinformatics tools, data workflows, and advanced statistical analyses are critical for managing complex datasets, facilitating mapping metabolites to curated databases and biological functions, and deriving meaningful insights. Future developments will depend on the integration of metabolomics with other omics technologies and bioassays to clarify regulatory mechanisms and biological functions. Approaches such as ML and network pharmacology can enhance structural validation, guide synthesis, and help bridge gaps between demand and extraction, including the exploitation of non-food sources such as vegetable waste and microalgae. The adoption of standardized protocols, annotation frameworks, and high-throughput bioactivity screening will further improve analytical reliability and data interpretation. Additionally, the validation of specific polyphenol biomarkers through human clinical trials is essential to define effective dosages for nutraceutical applications. Progress in spatial-temporal metabolomics and plant cell culture systems, combined with the exploration of underutilized indigenous species, particularly from regions such as Sub-Saharan Africa, will accelerate the discovery of novel polyphenols. Furthermore, adherence to FAIR (Findable, Accessible, Interoperable, and Reusable) data principles is vital to support data sharing, enable cross-study comparisons and facilitate the translation of metabolomics findings into functional foods development and therapeutic interventions targeting current and emerging health challenges.

## Figures and Tables

**Table 1:** Nutraceutical potential of selected plant materials.

Plant	Phytochemicals	Nutraceutical Potential	References
*F. abyssinica* extracts	Quercetin-rutinoside and sinapate derivatives	Antioxidant and antidiabetic	[7]
	Anthocyanin and stilbenoid-rich extracts	Antioxidant and anti-inflammatory	
	Quercetin, kaempferol glycosides	Anticancer or cardioprotective	
Mulberry (*Morus alba* L.) leaf extract	Quercetin, kaempferol, and their derivatives	Anticancer, neuroprotective, anti-inflammatory, and antidiabetic	[45]
Brown seaweeds in vitro	Flavonoids and phenolic acids	Antioxidant	[43]
*Garcinia subfalcata,* edible species	Flavonoid glycosides, phenolic acids, flavans, O-methylated flavonoids, linoleic acids, terpene glycosides, and triterpenoid saponins	Anticancer	[46]
*Annona muricata* L. (Leaf)	Alkaloids, flavonoids, and acetogenins	Anticancer	[47,48]
*Hibiscus sabdariffa* L. (Malvaceae)	Anthocyanins, flavonoids, aliphatic and phenolic acids	Alzheimer’s disease treatment	[40]
Wen-Shen-Yang-Gan decoction	Flavonoids, aliphatic and phenolic acids	Parkinson’s disease treatment	[42,49]
*Citrus aurantium* (unripe fruits and leaf) ethanolic extracts	7-trihydroxyflavone, isorhainetin, vitexin, and apigenin, and apigenin	Neuroprotective	[50]
*Opuntia ficus*-indica red fruit (OFI-RF) ethanol extracts	Chlorogenic acid and caffeic acid	Antidiabetic and anti-hypercholesterolemic	[51]
*B. cycloptera* fractions	Flavonoids, cardenolides, and phenolic acids	Antioxidant, anti-inflammatory, and antidiabetic	[52]
*Caryopteris mongolica* Bunge tea	Phenolic acids	Anti-rheumatoid arthritis	[53]
*Ludwigia adscendens* subsp. diffusa (Forssk.) P.H. Raven	Gallic acid, quercetin, ellagic acid, and betulinic acid	Antidiabetic, antioxidant, and anti-inflammatory	[54]
Red cabbage and broccoli seeds and sprouts	Amino acids and phenolic compounds	Antidiabetic	[55]

**Table 2:** Metabolomics techniques for the identification and quantification of polyphenols.

Technique	Plant and Part	Polyphenols and Others	References
GC-MS	*Combretum platypetalum*	Terpenoids, flavonoids	[28]
	*F. abyssinica*	Stilbenoids, lignans, coumarins, and complex tannins	[7]
UHPLC-HS-SPME/GC-MS	*Eleutherococcus senticosus* (Rupr. et Maxim.) (fruit)	Polyphenols (eleutherosides B, E, E1) and phenolic acids	[72]
UHPLC-HRMS	*Phlomis* species	Polyphenols, flavonoids, tannin, phenylalanine ammonia-lyase activity, photosynthetic pigments, and ascorbic acid levels	[65]
		Cinnamic acids, phenolic acids derived from galloyl quinic and shikimic acid, proanthocyanidins, glycosylated flavonoids, and triterpenes	[31]
	Aged black garlic	Polyphenols and anthocyanins	[39]
UHPLC-Q-TOF-MS2	Fermented *Perilla frutescens*	Apigenin, p-coumaric acid, rosmarinic acid, caffeic acid, polygallic acid, phenprobamate, hydroxy acetophenone, allopurinol, homovanillic acid, danshensu, and N-malayamycin	[47]
GC-MS, LC-QTOF-MS/MS	Quinoa (grains)	Flavonoid glycosides and saponins	[62]
	*Cajanus scarabaeoides*	Flavonoids and polyphenols	[73]
	*Selaginella*	Biflavonoids	[61]
	*Uapaca togoensis* (Leaf and stem bark)	Polyphenols	[42]
LC-HR-ESI-MS	*Bignonia binata* (Leaf)	Phenylethanoids, flavonoid glycosides, and iridoids	[74]
GC-MS/FT-IR	*Aporosa cardiosperma* (Leaf)	Flavonoid, phenol, and tannin	[29]
LC-MS and MALDI-MSI	*Fagopyrum tataricum* (L.) Gaertn. (Tartary Buckwheat) (various parts)	Phenolic acids and flavonoid. Flavonol glycosides and aglycones (in the embryo) and methylated flavonols, and procyanidins (in the hull)	[71]
UHPLC-QTOF-MS/MS	*Cydonia oblonga* Mill. (fruit)	Anthocyanins, flavones, flavones, flavan-3-ols, and flavonols, hydroxycinnamics, hydroxybenzoics, tyrosol, and stilbenes	[38]
	Shuang Huang Lian (SHL) (*Lonicerae japonicae* Flos, *Forsythiae fructus*, and *Scutellariae radix*)	Flavonoids, terpenoids, glycosylglycerol derivatives	[75]
	*Gliricidia sepium* (Jacq.) Kunth. ex Walp (Leaf)	Flavonoids, phenolic acids, triterpenoid saponins, fatty acid derivatives, and coumarins. Kaempferol-3-O-robinoside-7-O-rhamnoside, soyasaponin I & III, and 16-hydroxyhexadecanoic acid (major constituents	[56]
UHPLC-Q-Exactive Orbitrap MS	*Cocculus orbiculatus* (L.) DC. (dried roots, stem and flower)	Alkaloids, flavonoids, and organic acids	[76]
UHPLC-DAD-ESI-IT-TOF-MS^n^	*Astragali radix* plant	Polyphenols	[77]
HPLC-UV/DAD	*Amaranthus cruentus*	Quercetin, kaempferol, catechin, hesperetin, naringenin, hesperidin, and naringin, p-coumaric acid, ferulic acid, and caffeic acid, vanillic acid, and 4-hydroxybenzoic acid	[78]
UPLC-MS	Soybean varieties and cultivation sites (leaf)	Isoflavones, quercetin derivatives, and flavonol	[79]
UPLC-MS-NMR	*Crescentia cujete* (fruit pulp)	n-alkyl glycosides, phenolic acid derivatives (such as cinnamoyl and benzoyl derivatives), flavonoids, phenylethanoid derivatives, and iridoid glycosides	[19]

**Table 3:** Comparison of metabolomics platforms for polyphenols.

Technique	Accuracy	Sensitivity	Coverage	Quantitation Capability	References
UHPLC-MS (e.g., UHPLC-QTOF-MS, UPLC-ESI-HRMS)	High; excellent mass accuracy (e.g., <5 ppm with HRMS) and reproducibility due to fast gradients and stable ionization	Very high; low limit of quantification (LOQ: ng/mL range) via ESI and nano-flow options, ideal for trace polyphenols	Broad; untargeted profiling of diverse polyphenols (flavonoids, tannins) across polarity ranges	Strong for relative quantitation (e.g., via standards or isotopes); absolute needs calibration but handles matrix effects well	[80]
HPLC-MS (e.g., RP-HPLC-ESI-MS)	Good; reliable for targeted analysis but lower resolution than UPLC leads to co-elution risks	Moderate to high; LOQs in μg/mL, less optimal for ultra-trace than UPLC	Moderate; suits semi-targeted polyphenols but misses volatiles or isomers without HRMS	Excellent for targeted absolute quantitation with standards; cost-effective for routine use	[5,42,68]
GC-MS (e.g., HS-SPME/GC-MS, targeted GC-MS)	High for volatiles/derivatized phenolics; precise retention indices reduce false positives	Moderate; requires derivatization, limits non-volatiles (LOQs ~μg/g)	Narrow; best for small phenolics/acids, poor for glycosides or high-MW polyphenols	Good for absolute quantitation post-derivatization; reproducible but labor-intensive	[5,28,29]
LC-NMR (or hyphenated with MS)	Moderate; structural confirmation via 1D/2D spectra but lower precision in complex matrices	Low; poor for trace levels (mg/mL range) due to solvent suppression needs	Good for structural isomers; limited throughput	Poor; mainly qualitative, not routine for quantitation	[18,19,31]
Other (e.g., MALDI-MSI, IC-MS)	Variable; high spatial accuracy in imaging but matrix-dependent	High in localized analysis; not bulk-sensitive	Specialized (e.g., spatial metabolomes); narrow for polyphenols	Limited; relative only	[71]

**Table 4:** Data and pathway enrichment analysis in metabolomics.

Metabolomics + Plant Studied	Data Analysis	Pathway Analysis	Key Features	References
GC-MS (Untargeted)—*Combretum platypetalum*	Preprocessing by MetabR, PCA, HCA, multivariate analysis	Not reported	Bioactive metabolites and classified chemical profiles	[28]
GC-MS/FT-IR—*Aporosa cardiosperma (Gaertn.) Merr.*	PCA, compound library matching	Not reported	Profiled metabolites and linked to therapeutic potential	[29]
UHPLC-QTOF-MS (untargeted)—*Perilla frutescens*	PCA, OPLS-DA	Kyoto Encyclopedia of Genes and Genomes (KEGG)	Fermentation-induced bioactive metabolites with anticancer/immunomodulatory effects	[47]
UHPLC-Q-Orbitrap HRMS (untargeted)—*Annona muricata*	PCA, OPLS-DA	Not reported	Cytotoxic compounds active on MCF-7 cells	[48]
UPLC-MS/MS molecular networking—*Crescentia cujete (Bignoniaceae)*	GNPS molecular networking, clustering	Not reported	Structural annotation of untargeted phytochemicals	[19]
UPLC-qTOF-MS metabolite fingerprinting—*Macrotyloma geocarpum*	PCA, HCA	Not reported	Nutraceutical and antioxidant metabolite profiling	[67]
UHPLC-QTOF-MS—*Ocimum microgreens*	Multivariate analysis (PCA)	Not reported	Compared growing conditions and phenolic diversity	[41]
UPLC-MS/MS + chemometrics—*Bienertia cycloptera*	PCA, PLS-DA, chemometrics	Not reported	Anti-inflammatory fractions	[52]
UPLC-HRMS—*Ludwigia adscendens*	Multivariate analysis (PCA, OPLS-DA)	Not reported	Anti-inflammatory fractions	[54]
LC-MS + MALDI-MSI—Tartary buckwheat	PCA, spatial metabolomics analysis	KEGG	Spatial-temporal metabolite profiling during achene development	[71]
UHPLC-MS—*Halogeton glomeratus*	PCA, PLS-DA, OPLS-DA	KEGG	Metabolites linked to abiotic stress tolerance	[68]
HPLC + HRMS/MS + network pharmacology—*Sarcandra glabra*	OPLS-DA, pathway enrichment	KEGG + disease pathway mapping	Mechanisms in immune thrombocytopenia	[34]
UPLC-HESI (Untargeted) + network pharmacology—*Ornamental Camellia* flowers	PCA, OPLS-DA, network pharmacology	Gene Ontology (GO) + KEGG enrichment analysis	Bioactive metabolites and medicinal pathways	[23]
GC-MS + LC-QTOF-MS/MS + molecular networking—Quinoa	Chemometrics, molecular networking	KEGG	Anti-Alzheimer compounds; geographic variation	[62]
UPLC-Q-Orbitrap HRMS—Black garlic	PCA, OPLS-DA	KEGG	Biochemical changes during aging	[39]
UPLC-Orbitrap-MS/MS—Gallic acid intervention	PCA, correlation network analysis	KEGG	Linked polyphenols to lipid metabolism	[64]
LC-MS (Untargeted) + in silico screening—*Sisymbrium officinale*	PCA, docking, bioinformatics	Not reported	Flavonoid glycosides as anti-inflammatory agents	[37]
UPLC-ESI-MS/MS + signaling pathway study—*Citrus aurantium*	PCA, differential metabolite analysis	Not reported	Neuroprotection via signaling pathway modulation	[50]
UPLC/HESI-MS/MS—*Opuntia ficus-indica*	Multivariate analysis	Not reported	Phenolics & betanin preventing diabetic complications	[51]

**Table 5:** Data prerequisites, result interpretation and application scope.

Tools	Data Requirements	Result Interpretation	Scope of Application	References
MetaboAnalyst 6.0	LC-MS raw spectra in open formats (e.g., mzML) or pre-processed CSV/TSV peak-intensity tables with numerical values, sample-class labels (such control vs. treatment), and samples arranged in rows or columns; optional metadata table for multi-factor/time-series designs	Spectrum processing, multivariate statistics (PCA, PLS-DA, OPLS-DA), univariate tests, pathway-enrichment maps (such as metabolite set enrichment analysis (MSEA)/MetPA-style results), and causal-analysis modules are all available; users can interpret these results to find discriminating metabolites, enriched pathways, and potentially causative metabolite-phenotype relationships	Plant-based metabolomics research and biomarker-discovery applications can benefit from an all-inclusive web-based platform for targeted and untargeted metabolomics, which includes LC-MS, exposomics, and integration with pathway-enrichment and causal-inference procedures	[81,83,84,85]
Thermo compound discoverer	In-house databases (such as mzCloud and ChemSpider) are utilized for compound-annotation procedures; raw LC-MS/MS, GC-MS, or HRMS data files (such as Thermo Raw files) and sample-grouping metadata (factor levels, replicates) uploaded into the research software	Creates lists of known and unknown compounds, volcano-style graphs, and feature-level tables (*m*/*z*, RT, adducts, fragment-ions, annotation-match quality); users examine these tables to rank putative metabolites, improve annotations, and direct orthogonal validation (e.g., NMR)	Plant metabolomics, nutraceutical profiling, and exposomics all make extensive use of integrated software for small-molecule identification and characterization in complicated matrices (foods, plant extracts, biofluids)	[7]
MetPA	A metabolite concentration table with phenotypic labels (e.g., diseased vs. control, treated vs. untreated) or a collection of statistically significant metabolite identifiers (common names, KEGG and HMDB identifiers)	Produces ranked routes with *p*-values and topological “impact” scores using pathway enrichment and PT-based analysis; pathway map views show dysregulated nodes, assisting users in determining which metabolic modules are most severely disrupted	A specialized web-based metabolomics pathway analysis tool that connects metabolite lists to KEGG-compatible pathways and supports PT-based interpretation and overrepresentation in metabolomics studies focused on plants and diseases	[30]
Metscape	A separate gene-level table (such as Entrez identifiers) with related statistics (such as *p*-values and fold-change) for multi-omics integration and a metabolite-intensity table (CSV) with metabolite identifiers (such as KEGG-style identifiers) across samples	Allows users to explore metabolic-transcriptional linkages and deduce regulatory hubs that underlie observed metabolite patterns by interpreting network layouts that visualize metabolite-metabolite and metabolite-gene networks where node color/size encodes importance or fold-change	Pathway-centric and network-biology studies of plant-based or disease-related metabolite profiles are made possible by the cytoscape-based application that integrates metabolomics with transcriptome or proteomic data	[85]
Mummichog	A tab-delimited text file having columns for *m*/*z*, retention time, *p*-value (or significance), and test statistic (such as t-score) and one line for each *m*/*z* feature	Generates ranked routes with highlighted metabolites and enzymes by calculating pathway-enrichment scores from the feature list. Users may utilize these rankings to determine which biochemical pathways are probably changed, even in cases where many features lack reliable structural identifiers	Specifically created for untargeted metabolomics or other omics data where many measured entities are not fully annotated. Pathway and network analysis tool that facilitates exploratory analysis of intricately complex datasets connected to plants and diseases	[82,83,84,85]
KEGG	A list of metabolite or enzyme identifiers (such as gene numbers, KEGG Orthology (KOs) or KEGG compound identifiers) mapped from transcriptomic or metabolomic data; these are usually obtained from previous statistical analysis and metabolite–gene mapping	Users can locate dysregulated reactions and deduce functional modules implicated in a certain phenotype like stress-response, nutraceutical mechanism by annotating pathway maps with highlighted nodes based on fold-change, significance, or enrichment scores	In plant and clinical metabolomics, a central database of metabolic and signaling pathways is widely utilized as the foundation for pathway enrichment and PT-based tools (MetPA, MetaboAnalyst, etc.)	[23,30,84,85,86]
Gene Ontology (GO)	The input is usually a list of metabolites or genes with related statistics. A background set of all measured entities and a list of important metabolites or related genes mapped to GO keywords (for example, using Gene Ontology Analysis by the Integrated Data Science Laboratory for Metabolomics and Exposomics (IDSL.GOA)-style mappings	Beyond traditional pathway-map annotations, enriched GO terms (biological processes, molecular functions, cellular components) with *p*-values and term-specific scores are interpreted to capture broad functional themes (e.g., oxidoreductase activity, response to oxidative stress)	A functional annotation approach that supports pathway-based interpretation which is especially helpful when integrative multi-omics (metabolome plus transcriptome) study is being undertaken or when pathway-database coverage is restricted	[23,84]
Functional Class Scoring (FCS)	A set of pathway/gene-set definitions (e.g., KEGG-based or user-defined sets) and a full-ranked list of all measurable entities (e.g., metabolites or genes) arranged by a continuous metric (fold-change, correlation, or test statistic); no explicit binary differential-expression step is required	Enables users to identify pathways with weak per-metabolite impacts that over-representation analysis could overlook by producing enrichment-score plots and *p*-values for pathways that show subtle but coordinated shifts across functionally related entities	In metabolomics and multiomics, FCS-based techniques (such as MSEA-style methods in MetaboAnalyst) are employed as sensitivity-enhanced substitutes for ORA, particularly when polygenic or small-effect contributions are suspected	[84]
Pathway topology (PT)	A quantitative table (fold-change, *p*-values, or test scores) for each node in the pathway-network structure (nodes = metabolites/genes, edges = reactions/interactions) derived from KEGG-style databases; the PT-based modules are frequently integrated into pathway-analysis tools (MetPA, MetaboAnalyst, NetGSA-style tools)	Enables the interpretation of which pathways are both enriched and functionally central in the reported phenotype by providing topology-aware pathway scores that weigh metabolites according to their network role (hubs, bottlenecks)	By taking regulatory architecture and pathway connectivity into consideration, PT-based techniques are used to improve detection power in metabolomics and related omics	[30,84]

## Data Availability

No new data were created or analyzed in this study. Data sharing is not applicable to this article.
